# Maternal Methyl Donor Supplementation during Gestation Counteracts the Bisphenol A-Induced Impairment of Intestinal Morphology, Disaccharidase Activity, and Nutrient Transporters Gene Expression in Newborn and Weaning Pigs

**DOI:** 10.3390/nu9050423

**Published:** 2017-04-26

**Authors:** Hong Liu, Jun Wang, Daolin Mou, Lianqiang Che, Zhengfeng Fang, Bin Feng, Yan Lin, Shengyu Xu, Jian Li, De Wu

**Affiliations:** Institute of Animal Nutrition, Sichuan Agricultural University, No. 211, Huimin Road, Wenjiang District, Chengdu 611130, China; dkbshliuhong@sina.com (H.L.); wjss1987@163.com (J.W.); mdl19920101@foxmail.com (D.M.); clianqiang@hotmail.com (L.C.); fangzhengfeng@hotmail.com (Z.F.); fengb123d@163.com (B.F.); able588@163.com (Y.L.); shengyu_x@hotmail.com (S.X.); lijian522@hotmail.com (J.L.)

**Keywords:** bisphenol A, methyl donor, maternal, offspring, intestine

## Abstract

This study was conducted to explore whether exposure to bisphenol A (BPA) during pregnancy could change intestinal digestion and absorption function in offspring using pigs as a model, and whether methyl donor (MET) could counteract the BPA-induced impacts. Fifty Landrace × Yorkshire sows were divided into four dietary groups throughout gestation: control diet (CON); control diet supplemented with BPA (50 mg/kg); control diet supplemented with MET (3 g/kg betaine, 400 mg/kg choline, 150 μg/kg vitamin B12, and 15 mg/kg folic acid); and control diet with BPA and MET supplementation (BPA + MET). Intestine samples were collected from pigs’ offspring at birth and weaning. Maternal BPA exposure during pregnancy significantly reduced the ratio of jejunum villus height to crypt depth, decreased the jejunum sucrase activity, down-regulated the mRNA expression of jejunum peptide transporter 1 (*Pept1*) and DNA methyl transferase 3a (*DNMT3a*), and decreased the DNA methylation level of jejunum *Pept1* in offspring (*p* < 0.05). Maternal MET supplementation significantly raised the ratio of villus height to crypt depth in jejunum and ileum, improved the jejunum lactase activity, up-regulated the mRNA expression of jejunum *Pept1*, lactase (*LCT*), *DNMT1*, *DNMT3a*, and methylenetetrahydrofolate reductase (*MTHFR*), and increased the DNA methylation level of jejunum *Pept1* in offspring (*p* < 0.05). However, the ratio of jejunum villus height to crypt depth was higher in BPA + MET treatment compared with CON and BPA treatment (*p* < 0.05). Meanwhile, there was no difference in the jejunum sucrase activity, the mRNA expression of jejunum *Pept1* and *DNMT3a*, and the DNA methylation level of jejunum *Pept1* between CON and BPA + MET treatment. These results indicated that maternal exposure to BPA during gestation might suppress offspring’s intestinal digestion and absorption function, whereas supplementation of MET could counteract these damages, which might be associated with DNA methylation.

## 1. Introduction

Bisphenol A (BPA) is a kind of industrial chemical compound widely used in the manufacturing of polycarbonate plastics, such as baby bottles, infant tableware and the epoxy resin lining of beverage or food cans [1,2]. Exposure to BPA is a threat to human health according to the findings of its widely negative effects in animal studies [3]. As the prevalent existence of BPA in the environment as well as the detection in urine, plasma and placenta tissue of pregnant women [4], increasing evidence has shown that prenatal BPA exposure increased the risk of situations such as immune dysregulation, productive disorders, and disordered sexual differentiation [5,6]. To our knowledge, growth and health throughout the life cycle of mammals are closely related to their intestinal function, but the impact of gestational BPA exposure on the intestinal digestion and absorption function remains an unexplored endpoint. 

A growing body of research shows that a broad range of adverse effects induced by BPA are linked to epigenetic modifications, such as DNA methylation [7,8]. The first convincing study on agouti confirmed that maternal BPA exposure led to DNA methylation status and expression of specific altered genes in offspring [9]. An in vivo study has shown that BPA exposure decreased the global DNA methylation of oocyte, likely through decreased DNA methyltransferases [10]. Later evidence has demonstrated that gestational exposure to BPA decreased the global DNA methylation or methylation at the promoter in special genes in the brain, liver, and ovary tissue of the offspring [11,12]. Importantly, abnormal DNA methylation is associated with embryonic death, impaired fetal development, cancer and diseases of the brain, liver, kidneys and heart [13]. Therefore, researchers hypothesized that if BPA-induced intestinal function changes as speculated, it may be related to DNA methylation. DNA methylation is highly dependent on one-carbon metabolism, which involves sources of methyl groups such as choline, betaine and folate, as well as co-factors, for instance, vitamin B12 [14,15]. Therefore, methyl donor (MET) and co-factors from diet are crucial for normal DNA methylation. In mouse models, maternal diets containing much higher amounts of MET promoted DNA hypermethylation and led to long-term changes in gene expression and altered phenotypes in the offspring [16]. Even so, studies regarding the influence of maternal MET supplementation on intestine function in offspring are few. In this study, the effect of maternal MET supplementation was explored on intestinal function in offspring.

It is interesting that early developmental exposure to BPA can change offspring phenotype, stably altering the epigenome, but is counteracted by maternal MET supplements [9]. Therefore, this study explored the effects of maternal MET or BPA supplementation on intestinal function in offspring, and observed whether the effects last from birth to weaning.

## 2. Materials and Methods

### 2.1. Animals and Experimental Design

The experimental procedures were approved by the Animal Care and Use Committee of Animal Nutrition Institute, Sichuan Agriculture University, and followed the current laws of animal protection (Ethic Approval Code: SCAUAC201408-3). A total of fifty 3–5 parity Landrace × Yorkshire sows were artificially inseminated, then weighed (211.63 ± 2.65 kg), and randomly allocated to four groups at day 1 of gestation. Pregnant sows were fed one of the four diets: control diet (CON, *n* = 13); control diet + BPA (*n* = 13); control diet + MET (*n* = 12); and both BPA and MET supplementation in control diet (BPA + MET, *n* = 12). The control diet was the basal diet during pregnancy (Table 1), which completely met the recommendations of the National Research Council 2012 (NRC 2012), and nutritional values referenced from Chinese feed ingredients data (2013). The basal diet during pregnancy contained 3.05 Mcal digestible energy (DE)/kg diet and 14% crude protein. The BPA diet was supplemented with 50 mg/kg diet of BPA (Sigma Aldrich, St. Louis, MO, USA). MET supplementation premix consisted of 3 g/kg diet of betaine (98% betaine hydrochlorides; Hangzhou Healthy Husbandry Sci-tech Co., Ltd., Hangzhou, China), 400mg/kg diet of choline (50% choline chloride; Shandong Enbei Group Ltd., Shandong, China), 15 mg/kg diet of folate (Sigma Aldrich, St. Louis, MO, USA) and 150 μg/kg diet of vitamin B12 (Sigma Aldrich, St. Louis, MO, USA). The basal diet consisted of 482 g/kg diet of betaine, 1250 mg/kg diet of choline, 1.3 mg/kg diet of folate, and 15 μg/kg diet of vitamin B12, which were calculated values and met the recommended levels of choline, folate, and vitamin B12 (NRC, 2012). During lactation, all the sows were offered the same standard lactation diet, which contained 3.35 Mcal DE/kg diet and 17.5% crude protein. Lactation lasted 28 days. The experiment began with 56 sows–14 sows per treatment. Four sows were eliminated due to failure of gravidity, two sows were eliminated due to postpartum paralysis.

### 2.2. The Dose of Methyl Donors and BPA 

The criteria for the addition of doses of folic acid, betaine, choline and VB12 in this trial were designed to ensure the maximum potential reproduction of the sow and induce the reduction of homocysteine level in plasma. Previous studies found that dietary supplementation with 15 mg/kg of folic acid [17], 3 g/kg of betaine [18], 1650 mg/kg choline [19,20] or 150 μg/kg VB12 [21] was effective in increasing sow litter size, and significantly reduced homocysteine levels in plasma.

The dose of bisphenol A added was 50 mg/kg. Dolinoy (2007) [9] and other studies [8,22] in mice found that 50 mg/kg diet of BPA can successfully induce hypomethylation in a single gene or the entire organism. Therefore, in this article, the dose of BPA was calculated through the daily intake of unit weight of mice.

During gestation, all sows were individually fed their trial diets two times per day (i.e., 08:00 and 14:30 h): 2.28 kg/day diet and 2.72 kg/day diet on days 1–90 and days 91 to parturition, respectively. At day 107 of gestation, sows were moved to the farrowing rooms and individually penned. After parturition, all the sows were offered the diet three times per day (i.e., 08:00, 12:00, and 15:00 h), starting at 2.0 kg/day, then gradually increasing 1 kg/day until the sixth day ad libitum. Throughout the experiment, all animals were free to access water. The ambient temperature for sows was maintained at 20–25 °C. Heating light and pads were provided for sucking piglets and the temperature was maintained at 26–32 °C, which gradually decreased with the increase of age.

### 2.3. Sample Collection

When the piglets were born and weaning, researchers recorded the body weight of each, then selected piglets to be slaughtered and sampled according to body weight (body weight of newborn and weaning pigs seen in Table S1). Each group of 12 newborn piglets without eating colostrum (respectively from 12 litters and close to the average body weight in each treatment group, six males and six females) were slaughtered (a total of 48). Similarly, each group of six weaning piglets deprived of food overnight (respectively from six litters and close to the average body weight in each treatment group, three males and three females) were slaughtered (a total of 24). Piglets were slaughtered by jugular puncture, and the entire intestine was removed as quickly as possible. The content of the intestine was cleaned rapidly and gently. The small intestine (SI) was measured for weight and length. The middle portion (approximately four centimeters) of every segment in the small intestine (duodenum, jejunum and ileum) was obtained and placed in liquid nitrogen, later stored at −80 °C for subsequent analysis. In addition, approximately one centimeter of integral duodenum, jejunum and ileum tissue was cut out, and fixed with four percent paraformaldehyde for intestinal slice production and morphology observation. The intestinal mucosa of the remaining duodenum, jejunum, and ileum segment was obtained with a glass slide scraping, frozen in liquid nitrogen, and stored at −80 °C for future analysis of intestinal enzyme activity.

### 2.4. Analysis of Intestinal Morphology

Intestinal tissue samples were fixed with 4% formaldehyde, dehydrated with ethanol (from low concentration to high concentration up to 100%), and then embedded in transparent paraffin and sliced. All tissue sections (5 μm) were stained with hematoxylin and eosin (HE), and mounted with gum. The slice images were captured at the magnification of 100× using an Olympus BX51 microscope equipped with a DP70 digital camera (Olympus, Tokyo, Japan). Crypt depth (μm) and villus length (μm) were measured with Image-Pro Plus 6.0 software (Media Cybernetics, Bethesda, Rockville, MD, USA). At least eight pairs of villus and crypt were observed in each slice; the average was the final value.

### 2.5. Analysis of Intestinal Enzyme Activity

The activity of intestinal lactase, maltase, and sucrase was measured. After thawing, 0.2–0.5 g of mucosa samples was homogenized according to the weight of intestine mucosa (g): volume of physiological saline pre-cooled on ice (mL) = 1:9, and centrifuged at 2500 r/min for 10 min. The supernatant was used to measure activity. Before measurement, a pre-test was necessary for the optimal reaction condition. For instance, the appropriate reaction concentration of samples. The activity of disaccharide was measured strictly in accordance with the manufacturer’s instructions for the kit (Jiancheng Bioengineering Ltd., Nanjing, China).

### 2.6. RT-PCR Analysis

Jejunum samples stored in a −80 °C freezer were ground to powder with a mortar, adding liquid nitrogen continually. A 50–100 mg powder sample was put in an eppendorf tube containing 1 mL TRIzol (Invitrogen, Carlsbad, CA, USA), shaken up and down so as to completely dissolve the powder. Total RNA was extracted with TRIzol. RNA quality was detected by means of electrophoresis with 1.0% agarose gel at low temperature, 80 V for 25 min, observing the picture under ultraviolet light. The strips should be clear and complete without smearing. The absorbance of RNA solution at wavelengths of 260 nm and 280 nm was measured by scanning spectrophotometer (Beckman DU-800, Beckman Coulter Inc., Brea, CA, USA); resulting in optical density (OD)_260_/OD_280_ ranging 1.8–2.0. The concentration of RNA solution was confirmed by nucleic-acid/protein analyzer (Beckman DU-800) to determine the required amount in the next reverse transcription. After that, cDNA was synthesized with a reverse transcription (RT) kit (TaKaRa Biotechnology, Dalian, China), according to the manufacturer’s instructions. The cDNA can be stored at −20 °C for later relative quantification by polymerase chain reaction (PCR) or immediately used. Real-time PCR was performed with the synergy brands (SYBR) green mix system (catalogue no. RR820A: TaKaRa Biotechnology, Dalian, China) by ABI-7900HT instrument (Applied Bio Systems, Foster City, CA, USA). Ten microliters of mixed system (5 μL SYBR Green Super mix, 1 μL upstream primer, 1 μL downstream primer, 0.2 μL Reference Dye II, and 2.8 μL cDNA diluted with ddH_2_O) was used for RT-PCR. Primer sequences were designed by Primer 5.0 and shown in Table 2. The conditions were as follows: pre-denaturation at 95 °C for 30 s, followed by 40 cycles of denaturation at 95 °C for 5 s and annealing at 60 °C for 34 s. The expression of *β-actin* was shown to be consistent, and was amplified in parallel with the target genes as an inner control. The optimal annealing temperature of each primer and the range of PCR cycles was determined before the formal determination. The correlation coefficients of all the standard curves were >0.99, and the amplification efficiency values were between 90% and 110%. At the end of target gene amplification, researchers performed a melting curve analysis to identify amplification specificity. The mRNA expression of target genes normalized with the house keeping gene *β-actin* was calculated with the 2^−ΔΔ*C*t^ method.

### 2.7. MassARRAY-Quantitative DNA Methylation Analysis

The results of nutrition transporters gene expression measurement showed that *Pept1* gene expression was significantly affected by the BPA and MET supplementation, and in view of its importance for oligopeptides transportation, this study determined the methylation status of the *Pept1* promoter regions. The Cytosine-phosphoric acid-Guanine (CpG)-rich sequences for the *Pept1* promoter regions were identified using the USCS genome browser, from 2000 bp upstream of the transcriptional start point. Four different EpiTYPER assays (sense strand 2, 10, 14 and 20) were designed (EpiDesigner), which covered 8, 8, 8 and 13 CpG sites respectively (successful data were generated for 6, 6, 7 and 6 CpG sites). The primer sequences and the locations relative to the translational start codon for the assays are presented in Table 3. Quantitative methylation analysis of the porcine *Pept1* promoter was performed using Sequenom’s MassARRAY EpiTYPER protocol (Sequenom, San Diego, CA, USA). Genomic DNA was extracted from the newborn piglets’ intestine using a Wizard Genomic DNA Purification Kit (Promega Corporation, Madison, WI, USA), and then bisulfite-treated with the EZ DNA Methylation Kit (Zymo Research, Orange, CA, USA). Specific PCR was carried out with the bisulfate-modified DNA. The conditions were as follows: 94 °C for four minutes followed by 45 cycles of 94 °C for 20 s, 52–62 °C for 30 s and 72 °C for three minutes. The PCR products were detected on agarose gel before the further analysis. In vitro transcription and RNase cleavage reaction were conducted using the Mass Cleave (hMC) kit. Transcription cleavage products were dispensed onto a 384 element SpectroCHIP bioassay, and the mass spectra were acquired through a MassARRAY mass spectrometer (Sequenom). The spectra were analyzed and the methylation rations were obtained by the EpiTYPER software v.1.0 (Sequenom). Each step of the above operation was performed in strict accordance with the corresponding manufacturer’s instructions.

### 2.8. Statistical Analysis

Raw data were preliminarily processed with MS Excel, with further data analysis performed by two-way ANOVA using SPSS Statistics 20 (IBM^®^ SPSS^®^ Statistics, New York, NY, USA). Data were tested for normality and homogeneity of variances (Shapiro–Wilk and Levene tests, respectively) and when necessary data were normalized (arcsine, square root or logarithm normalized) to achieve ANOVA assumptions. Additionally, Duncan’s multiple range tests were conducted to determine difference among the treatments. All data were expressed as mean and standard error. *p* < 0.05 was considered statistically significant.

## 3. Results

### 3.1. Intestinal Index

Maternal BPA exposure during gestation had no significant effect on the intestinal index. However, MET supplementation significantly decreased the ratio of SI length to body weight but increased the ratio of SI weight to length both in newborn and weaning piglets (*p* < 0.05) (Table 4).

### 3.2. Intestinal Morphology

Maternal BPA supplementation during gestation significantly reduced the ratio of jejunum villus height to crypt depth in newborn and weaning offspring (*p* < 0.05). Maternal MET supplementation significantly increased the jejunum villus height of newborn piglets, and the ratio of jejunum and ileum villus height to crypt depth both in newborn and weaning piglets (*p* < 0.05). Additionally, the ratio of jejunum villus height to crypt depth in newborn offspring and the ratio of ileum villus height to crypt depth in weaning offspring were significantly influenced by BPA × MET interaction (*p* < 0.05) (Table 5).

### 3.3. Disaccharidase Activity

Maternal BPA supplementation during gestation significantly decreased the activity of jejunum sucrase in offspring both at birth and weaning (*p* < 0.05). Maternal MET supplementation significantly improved the activity of jejunum lactase in both newborn and weaning pigs, and the activity of duodenum and jejunum sucrase in weaning piglets (*p* < 0.05). The activity of jejunum lactase in newborn and weaning pigs was significantly affected by BPA × MET interaction (*p* < 0.05) (Table 6).

### 3.4. Nutrient Transporter Gene Expression

Maternal BPA supplementation during gestation significantly down-regulated the mRNA relative expression of jejunum *Pept1* and *SUC* in newborn piglets (*p* < 0.05). Maternal MET supplementation significantly up-regulated the mRNA relative expression of jejunum *Pept1*, *Sglt1* and *LCT* in newborn offspring and weaning offspring. *SUC* was only affected at birth (*p* < 0.05). Compared with the BPA group, the mRNA relative expression of jejunum *Pept1* was significantly increased in the BPA + MET group at birth and weaning (*p* < 0.05) (Table 7).

### 3.5. CpG Methylation

**Figure 1:**
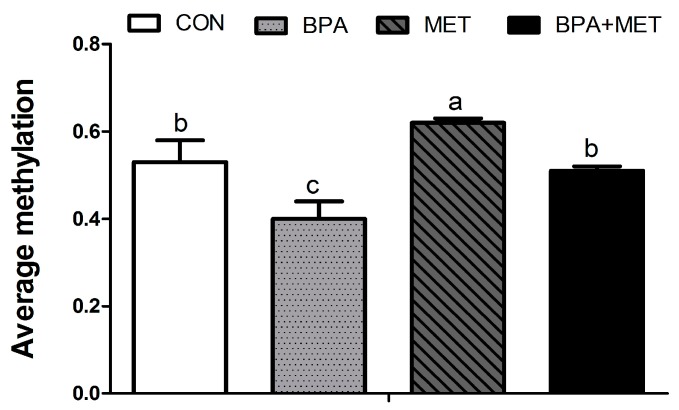
Effect of maternal methyl donor or bisphenol A supplementation during gestation on the jejunum *Pept1* gene DNA average methylation level in newborn pigs. CON, control; BPA, bisphenol A; MET, methyl donor; BPA + MET, both bisphenol A and methyl donor supplementation in control diet. Values are expressed as mean ± standard error; different superscript letters within a row indicate significant difference (*p* < 0.05). Four independent samples per treatment group (total 16).

In jejunum tissue of newborn pigs, maternal BPA exposure during gestation significantly reduced the methylation rate of the CpG site −18, −66, −93, −215, −688, −721, −1352, −1544, −1705 and−1771 on the *Pept1* promoter region (*p* < 0.05) (Table 8). Whereas the methylation rates of these CpG sites, except the −1771 site, were significantly increased by maternal MET supplementation (*p* < 0.05), the methylation rates of CpG sites −93 and −721 were significantly influenced by BPA × MET interaction (*p* < 0.05). Compared with the BPA group, the average methylation of CpG sites at jejunum *Pept1* promoter was significantly increased in the BPA + MET group (Figure 1).

### 3.6. Methylation-Related Gene Expression

**Figure 2:**
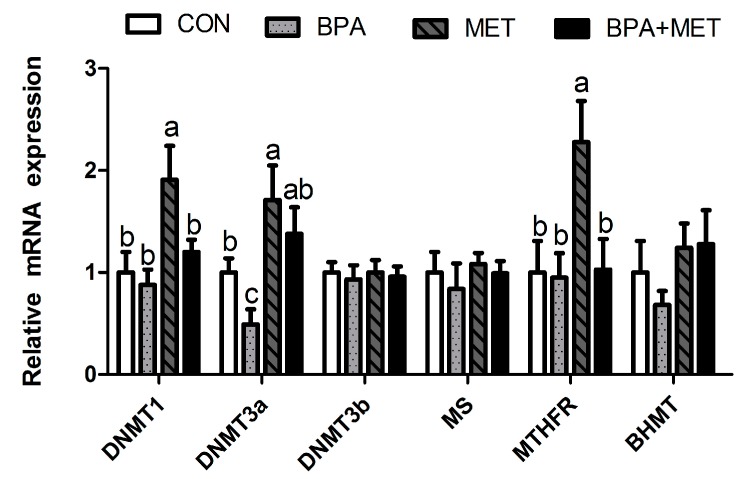
Effect of maternal methyl donor or bisphenol A supplementation during gestation on mRNA relative expression of key enzymes related to DNA methylation in jejunum of newborn piglets. CON, control; BPA, bisphenol A; MET, methyl donor; BPA + MET, both bisphenol A and methyl donor supplementation in control diet; *DNMT*, DNA methyl transferase; *MS*, methionine synthase; *MTHFR*, methylenetetrahydrofolate reductase; *BHMT*, betaine homocysteine methyltransferase. Data are normalized against *β-actin*, with results expressed relative to the control sample using the ^ΔΔ^*C*t method (where *C*t is the cycle threshold) with efficiency correction. Values are expressed as mean ± standard error; different superscript letters within a row indicate significant difference (*p* < 0.05). 12 independent samples per treatment group (total 48).

Maternal BPA supplementation during gestation significantly reduced the mRNA relative expression of jejunum *DNMT3a* in newborn offspring (*p* < 0.05). Maternal MET supplementation significantly increased the mRNA relative expression of jejunum *DNMT1*, *DNMT3a* and *MTHFR* in newborn piglets (*p* < 0.05). Compared with the BPA group, the mRNA relative expression of jejunum *DNMT3a* was significantly increased in the BPA + MET group at birth (*p* < 0.05) (Figure 2).

## 4. Discussion

This was the first study to focus on the effect of maternal BPA exposure during pregnancy on intestinal digestion and absorption function in offspring. In this study, the ratio of jejunum villus height to crypt depth in newborn and weaning pigs was significantly decreased by maternal BPA exposure. The ratio of villus height to crypt depth reflects the capacity of the small intestine for nutrient digestion and absorption to a certain extent [23]. The proliferation and differentiation of intestinal epithelial cells is accompanied by their migration along the crypt-villus axis [24]. Thus, a lower ratio of villus height to crypt depth may indicate less proliferation and differentiation. It is logical to consider that maternal BPA exposure may suppress digestion and absorption function through inhibiting the proliferation and differentiation of intestinal epithelial cells in offspring. Researchers have reported that prenatal exposure to BPA affected immune function in offspring; resulting in higher IFN-γ and IL-4, and lower CD4^+^ and CD25^+^ cells [5,25,26], which were closely related to the proliferation of epithelial cells [27,28]. Most mature intestine epithelial cells have the function of synthesis and secretion of digestive enzymes [29]. 

In this study, the jejunum sucrose activity in offspring was significantly decreased in response to BPA exposure, which was consistent with the *SUC* mRNA expression. Lactose is the main source of mammalian carbohydrate during lactation, so lactase is the dominant enzyme in the intestine of suckling piglets [30]. However, with the increase of age (especially after weaning), maltase and sucrase gradually become dominant enzymes. The results showed that the jejunal lactase in neonatal and weaned piglets was not significantly affected by BPA, but sucrose was. It implied that BPA exposure during pregnancy did not significantly affect the energy intake of newborn piglets, but might have an impact with increasing age. This may be the reason that the average body weight of piglets in the BPA group was not significantly different from that in the MET group at birth, but tended to be lower at weaning. However, this mechanism requires further study.

Consistently, the development of absorption function in the intestine relied greatly on the intestinal nutrient transporter [31]. Increasing intestinal absorption is generally linked to the elevated expression of nutrient transporters [32]. In this research, the jejunum *Pept1* mRNA level was observed to be significantly decreased in BPA-exposed offspring. Pept1 is located at the brush-border membrane of intestinal epithelial cells and mediates cellular uptake of these oligopeptides by using an inwardly directed H^+^ gradient across the brush-border membrane [33]. Therefore, the transfer and absorption of oligopeptides were closely related to the *Pept1* gene expression [34].

Recently, a growing number of studies showed changes in gene expression due to BPA, especially the long-term effects but at low does, possibly involving epigenetic pathways [35]. In this study, a significant decrease of the average methylation rate on the jejunum *Pept1* promoter region in newborn BPA-pigs was obtained. Researchers found that the CpG sites −1771 and −721, where the methylation rate were decreased, were located in the transcription factor cAMP-response element binding protein (CREB) binding sites, and CpG sites −1544, −1352 and−93 were located in the transcription factor stimulatory protein 1 (SP1) binding sites. Studies have demonstrated the importance of the transcription factors CREB and SP1 in up-regulation of *Pept1* expression [36,37]. Traditionally, many researchers tend to deem that CpG islands (CGIs) are mostly not methylated when located at the upstream promoter of transcription start sites. Once methylated by external environmental factors, methylated CGIs can result in stable repression of the linked gene, likely by preventing them from binding to transcription factors [38]. However, this study seemed inconsistent with this hypothesis. The most reasonable explanation is that there are just CpG sites, but no CGIs in the promoter of *Pept1*.The functional relevance of reduced methylation at CG-poor regulatory regions is still unclear. Although the related mechanisms remain unclear, this study indicated that *Pept1* gene expression decline via hypomethylation modifications was a mechanism by which the maternal BPA was not beneficial for oligopeptides transfer and absorption in offspring. It is known that *DNMT3a* is one of the DNA methyltransferases, catalyzing the formation of de novo methylation [39]. In this study, mRNA-relative expression of jejunum *DNMT3a* in BPA-exposed newborn pigs was significantly decreased, which indicated that BPA-induced hypomethylation was likely through decreased DNA methyltransferases. Research has shown that the effects of BPA were manifested in disrupted fetal metabolic programming through hypomethylation modifications via decreased DNMT, even though the concrete mechanisms remain unclear [40].

The intestinal morphology results suggested that MET could induce the increase of the intestinal epithelial cell proliferation as demonstrated by the increased ratio of villus height to crypt depth in MET offspring pigs. A similar study reported that the maternal MET (vitamin B12, folate and choline) deficiency during gestation and lactation in rats induced higher intestinal crypt apoptosis and lower villi epithelial cell differentiation in offspring [41]. Consistent with the intestinal morphology results, we found that jejunum mucosal lactase activity and the expression of the lactase gene were significantly increased in MET offspring, which meant a better digestion ability for lactose. Lactose is the major carbohydrate in milk of most placental mammals, and lactase plays a dominant role in digestion of disaccharide before weaning [30]. 

In this study, MET increased the *Pept1* and *Sglt1* genes expression in newborn pigs’ jejunum, which were positively correlated with transportation and absorption for oligopeptides and glucose. Studies have shown that the changes of gene expression resulted from MET were closely related to DNA methylation [42]. As expected, maternal MET supplementation increased the average methylation rate on the jejunum *Pept1* promoter region in newborn pigs. Among the 25 CpG sites tested on the *Pept1* promoter region, the methylation rate of eight CpG sites was significantly influenced by MET. The increased CpG site −721 was located in the transcription factor CREB binding sites; CpG sites −1544 and −93 were located in the transcription factor SP1 binding sites. Research has suggested that *Pept1* gene expression is mainly regulated by some transcription factor, such as caudal-related homeobox 2 (CDX2), CREB, amino acid response element (AARE) and SP1 [43]. Recently, genomic DNA methylation and transcriptome has shown a positive correlation of gene expression and methylation levels at some special genes’ promoter which was not located in CGI regions [44]. The transcription factor which was sensitive to methyl directly bound to the methylated CpG region to promote gene transcription when the methylation level of the low-density CpG region was increased. The transcription factor which was not sensitive to methyl as a leading transcription factor specifically bound to the methylated CpG region to promote the binding between the downstream methylation-sensitive transcript factor and this region, finally resulting in the transcript and expression. This study’s results were in agreement with studies on mice with maternal diets containing much higher amounts of methyl donors. Diets high in methyl donors promoted DNA hypermethylation and led to long-term changes in gene expression and altered phenotypes in the offspring [16]. 

DNA methylation occurs within the one-carbon metabolism pathway, which is dependent upon several important enzymes. Through an ATP-driven reaction with methionine adenosyltransferase (MAT), a carbon product, methionine, whose methyl groups are derived either from the folate/vitamin B12 pathway or choline/betaine pathway is converted into S-adenosylmethionine (SAM), the cellular direct methyl donor [45]. Then, DNA methyltransferases (DNMTs) covalently attach methyl groups from SAM to the carbon-5 position of cytosine bases, generating 5-methylcytosine, thus methylating DNA [42]. MTHFR and MS are key enzymes in the folate/vitamin B12 pathway and BHMT in the choline/betaine pathway [46]. Therefore, the up-regulation of jejunum *DNMT1*, *DNMT3a*, and *MTHFR* genes in MET newborn pigs in this study further confirmed that the higher *Pept1* genes expression might be related to DNA methylation. These results suggested that maternal MET supplementation could promote offspring’s intestinal digestion and absorption function by improving intestinal morphology, increasing disaccharidase activity, and up-regulating the expression of the transporter gene via methylation changes. 

It is noteworthy that intestinal morphology, disaccharidase activity and nutrient transporters gene expression in offspring were significantly influenced by the interaction of maternal BPA and MET treatment. Compared with the BPA group, the ratio of jejunum villus height to crypt depth in offspring, the jejunum lactase activity in newborns, the duodenum sucrose activity in weaning pigs, and the *Pept1* gene expression in offspring of the BPA + MET group were significantly higher. This indicates that MET could counteract the negative effects of BPA on intestinal digestion and absorption function. Moreover, these consequences could still persist until weaning, even if administration was not implemented during lactation. One possible reasonable explanation is epigenetic change, most likely DNA methylation according to a previous study on BPA or MET [8]. Once DNA methylation patterning is established, which can be stably inherited throughout the life course. The average methylation of CpG sites at the jejunum *Pept1* promoter was significantly increased in the BPA + MET group. However, the specific mechanism by which MET interacts with BPA through the DNA methylation pathway requires further study.

## 5. Conclusions

In conclusion, the results of this study indicated that maternal BPA supplementation during gestation might suppress offspring intestinal digestion and absorption function, and MET could counteract the damage, which might be related to the DNA methylation status of specific genes.

## Figures and Tables

**Table 1:** Ingredients and nutrient composition of basal diets fed to sows throughout gestation and lactation.

Item	Gestation	Lactation
Ingredients (g/kg)		
Corn	636.15	629.00
Soybean meal	145.00	226.00
Fish meal		25.00
Soybean oil		30.00
Wheat bran	180.00	50.00
L-lysine·HCl (98%)	0.80	2.70
D-Methionine (98%)		1.10
Calcium carbonate	11.10	10.00
Dicalcium phosphate	13.80	10.70
Sodium bicarbonate		4.00
Choline (50%)		1.00
Salt	3.00	4.00
Vitamin and Mineral Premix *	10.15	6.50
Total	1000.00	1000.00
Nutrient level ^†^		
Digestible energy, Mcal/kg	3.05	3.35
Crude protein, %	14.00	17.50
Total Lysine, %	0.69	1.12
Standard ileal digestible-Lysine, %	0.60	1.00
Total calcium, %	0.80	0.80
Total phosphorus, %	0.68	0.63

* Vitamin and Mineral mixture for gestation sows supplied the following amounts of vitamins/kg and minerals/kg of complete diet: 9100 IU vitamin A; 2600 IU vitamin D3; 80 IU vitamin E; 2.6 mg vitamin B1; 6.5 mg vitamin B2; 3.9 mg vitamin B6; 15 μg vitamin B12; 26 mg niacin; 1.3 mg folacin; 265 mg choline; 120 mg iron; 20 mg copper; 120 mg zinc; 30 mg manganese; 0.3 mg selenium; 0.3 mg iodine; 0.2 mg chromium. Vitamin and Mineral mixture for lactation sows supplied the following amounts of vitamins/kg and minerals/kg of complete diet: 17,500 IU vitamin A; 5000 IU vitamin D3; 80 IU vitamin E; 5 mg vitamin B1; 12.5 mg vitamin B2; 7.5 mg vitamin B6; 0.05 mg vitamin B12; 50 mg niacin; 25 mg pantothenic acid; 2.5 mg folacin; 120 mg iron; 20 mg copper; 120 mg zinc; 30 mg manganese; 0.3 mg selenium; 0.3 mg iodine; 0.2 mg chromium. ^†^ The basal diet for gestation sows contained: 482 mg/kg betaine, 1.3 mg/kg folic acid, 15 μg/kg VB12 and 1250 mg/kg choline calculated according to the China Feed Database (2014).

**Table 2:** Oligonucleotide primers used for a relative-quantitative real-time PCR analysis.

Genes	Gene Bank No.	Sequences (5′-3′)
*β-actin*	AY550069.1	Forward: CCAGCACGATGAAGATCAAGA Reverse: AATGCAACTAACAGTCCGCCTA
*Slc7a9*	NM_001110171.1	Forward: GAACCCAAGACCACAAATC Reverse: ACCCAGTGTCGCAAGAAT
*Pept1*	AY180903.1	Forward: GATGAAATGTGAGCGTATGGG Reverse: AAAGAGGGAGGATCTGGAAAA
*Sglt1*	NM_001164021.1	Forward: CCACTTTCCCTATAAAACCTCAC Reverse: CTCCATCAAACTTCCATCCTCAG
*Glut2*	NM_001097417.1	Forward:CCTGCTTGGTCTATCTGCTGTG Reverse:TTGATGCTTCTTCCCTTTCTTT
*LCT*	XM_003359430.4	Forward: TGTGCAGCGGTTTAAGGAGTAT Reverse: CCACAACAAAGGGCTCATTCAG
*SUC*	XM_013990124.1	Forward: TTATCCGACCCCTTTTGCATGA Reverse: CGAGCATTAGGGACATAGCCTT
*MGAM*	XM_005657730.2	Forward: AGGCATCCAATTCTTCTGGAGT Reverse: GGCCCCAAATGAGTCATACTGA
*DNMT1*	DQ060156.1	Forward: TTTCGTCTCCTTCAAGCGCT Reverse: CCATACTGACCAGCCTGCAA
*DNMT3a*	DQ785811.1	Forward: AGTGCGTGGATCTCTTGGTG Reverse: TCCTGGTCGTGGTTATTGGC
*DNMT3b*	NM_001162404.1	Forward: TGAAGAGTCCATCGCTGTTG Reverse: CAATCACCAGGTCAAAGGG
*MS*	AF276463	Forward: AGCTTTGTTCGCAGTCCAGA Reverse: AAGGTCTCATTTCGGCTGCA
*MTHFR*	AF239166	Forward: CAGTGGGAAGCAGAGGAAGG Reverse: GCCACACAGACGTCGAAGTA
*BHMT*	NM_001200042.1	Forward: GAGGCTGTGTGGGCAGTTGAAG Reverse: ACAATGGATGCTCCTGCCTTTACC

*Slc7a9*, amino acid transporter light chain, family 7, member 9; *Pept1*, peptide transporter 1; *Sglt1*, sodium/glucose cotransporter 1; *Glut2*, glucose transporter 2; *LCT*, lactase; *SUC*, sucrase-isomaltase (alpha-glucosidase); *MGAM*, maltase-glucoamylase; *DNMT*, DNA methyl transferase; *MS*, methionine synthase; *MTHFR*, methylenetetrahydrofolate reductase; *BHMT*, betainehomocysteine methyltransferase.

**Table 3:** The primer sequences and the locations relative to the translational start codon for the assays in MassARRAY-quantitative DNA methylation analysis.

Assays	Sequences (5′-3′)	Location
*Pept1*-2	Forward: GTGGGGTTAGATTTTTTTAAAATGG Reverse: AAAAAAAACCACATCCACAAATAAA	−968 to −1481
*Pept1*-10	Forward: GTTTATTTGGGTGGAGATTGTTTAG Reverse: ACCCTTAACCCAATAAATAAAACCA	−1450 to−1089
*Pept1*-14	Forward: AGTTTATATTTGGTGTGGTTGTGGT Reverse: CCCACCTCCCTATATTAACAAAAAA	−1050 to −599
*Pept1*-20	Forward: GTTGGGTGTTAGGTATTTTTAAGGG Reverse: AACAAAACCAACTATAAAACTCCCA	−390 to +75

*Pept1*, peptide transporter 1.

**Table 4:** Effect of maternal methyl donor or bisphenol A supplementation during gestation on intestinal index in newborn and weaning pigs.

	Treatment				*p*-Value		
	CON	BPA	MET	BPA + MET	BPA	MET	BPA × MET
Newborn							
Body weight, kg	1.27 ± 0.02 ^b^	1.38 ± 0.02 ^a^	1.39 ± 0.01 ^a^	1.38 ± 0.02 ^a^	0.04	0.01	0.01
SI (g)	41.77 ± 3.11	38.78 ± 3.02	42.76 ± 4.10	43.48 ± 3.12	0.70	0.33	0.53
SI (cm)	389.49 ± 21.02	371.37 ± 23.77	370.20 ± 19.54	356.05 ± 20.68	0.31	0.27	0.90
SI (g × kg^−1^ BW)	32.56 ± 2.09	28.08 ± 1.98	30.83 ± 3.37	31.37 ± 2.72	0.32	0.69	0.21
SI (cm × kg^−1^ BW)	306.27 ± 24.02 ^a^	269.39 ± 15.05 ^b^	266.32 ± 16.07 ^b^	258.22 ± 16.51 ^b^	0.06	0.03	0.20
SI weight/length (mg/cm)	106.09 ± 7.02 ^b^	104.35 ± 7.42 ^b^	116.14 ± 6.58 ^a^	122.80 ± 8.00 ^a^	0.69	0.02	0.49
Weaning							
Body weight, kg	6.54 ± 0.09 ^c^	7.01 ± 0.28 ^b^	7.54 ± 0.13 ^a^	7.13 ± 0.09 ^b^	0.86	0.00	0.01
SI (g)	190.18 ± 14.05	200.28 ± 13.99	220.62 ± 12.57	192.82 ± 13.78	0.77	0.62	0.44
SI (cm)	935.67 ± 45.44	954.38 ± 49.22	964.18 ± 39.49	958.73 ± 45.43	0.85	0.64	0.73
SI (g × kg^−1^ BW)	29.09 ± 3.22	29.85 ± 4.12	28.06 ± 5.13	28.39 ± 2.79	0.82	0.60	0.93
SI (cm × kg^−1^ BW)	143.25 ± 11.54 ^a^	138.89 ± 14.32 ^a^	126.91 ± 13.97 ^b^	134.32 ± 15.07 ^a,b^	0.81	0.05	0.35
SI weight/length (mg/cm)	203.47 ± 27.36 ^b^	215.85 ± 29.20 ^a,b^	231.30 ± 30.32 ^a^	221.60 ± 20.01 ^a^	0.93	0.04	0.50

CON, control; BPA, bisphenol A; MET, methyl donor; BPA + MET, both bisphenol A and methyl donor supplementation in control diet; SI, Small intestine. Different superscript letters within a row indicate significant difference (*p* < 0.05). At birth, 12 independent samples per treatment group (total 48); at weaning, six independent samples per treatment group (total 24).

**Table 5:** Effect of maternal methyl donor or bisphenol A supplementation during gestation on intestinal morphology in newborn and weaning pigs.

	Treatment				*p*-Value		
	CON	BPA	MET	BPA + MET	BPA	MET	BPA × MET
Newborn							
Duodenum							
Villus height (μm)	479.86 ± 33.57	442.69 ± 37.11	522.68 ± 39.52	512.70 ± 34.79	0.70	0.37	0.83
Crypt depth (μm)	125.57 ± 11.50	126.45 ± 13.47	127.25 ± 12.98	131.08 ± 14.01	0.78	0.71	0.86
Villus/crypt ratio	3.87 ± 0.43	3.50 ± 0.32	3.94 ± 0.27	4.12 ± 0.38	0.85	0.49	0.59
Jejunum							
Villus height (μm)	639.99 ± 59.30 ^b^	649.53 ± 61.37 ^b^	740.11 ± 60.54 ^a^	717.39 ± 55.83 ^a^	0.75	0.05	0.82
Crypt depth (μm)	104.73 ± 6.91	114.33 ± 7.12	102.18 ± 5.90	99.11 ± 7.01	0.61	0.17	0.33
Villus/crypt ratio	5.94 ± 0.62 ^b^	4.19 ± 0.57 ^c^	7.99 ± 0.52 ^a^	8.69 ± 0.71 ^a^	0.04	0.02	0.04
Ileum							
Villus height (μm)	579.19 ± 50.11	530.19 ± 53.25	620.89 ± 51.33	653.98 ± 52.17	0.92	0.31	0.61
Crypt depth (μm)	111.18 ± 13.29	122.27 ± 10.12	95.33 ± 9.02	127.51 ± 9.74	0.07	0.64	0.36
Villus/crypt ratio	5.58 ± 0.54 ^b^	5.19 ± 0.62 ^b^	6.45 ± 0.56 ^a^	5.86 ± 0.48 ^a,b^	0.86	0.03	0.75
Weaning							
Duodenum							
Villus height (μm)	287.91 ± 22.89	277.98 ± 25.37	324.57 ± 21.09	308.72 ± 19.88	0.73	0.36	0.94
Crypt depth (μm)	178.45 ± 10.30	171.55 ± 9.02	174.37 ± 11.06	183.46 ± 13.00	0.95	0.82	0.64
Villus/crypt ratio	1.74 ± 0.12	1.62 ± 0.22	1.96 ± 0.15	1.89 ± 0.12	0.61	0.20	0.90
Jejunum							
Villus height (μm)	264.71 ± 19.03	243.04 ± 21.24	331.47 ± 24.01	274.58 ± 20.59	0.27	0.17	0.61
Crypt depth (μm)	132.93 ± 9.77	142.21 ± 10.02	133.97 ± 8.29	120.26 ± 9.15	0.84	0.34	0.30
Villus/crypt ratio	2.04 ± 0.15 ^b^	1.51 ± 0.12 ^c^	2.54 ± 0.12 ^a^	2.60 ± 0.13 ^a^	0.02	0.03	0.48
Ileum							
Villus height (μm)	206.80 ± 39.56	233.29 ± 33.47	291.58 ± 36.90	314.50 ± 35.36	0.67	0.20	0.99
Crypt depth (μm)	113.45 ± 7.89	129.57 ± 8.03	122.36 ± 9.76	106.86 ± 12.04	0.98	0.60	0.24
Villus/crypt ratio	1.84 ± 0.08 ^b^	1.83 ± 0.08 ^b^	2.58 ± 0.09 ^a^	1.85 ± 0.010 ^b^	0.64	0.02	0.02

CON, control; BPA, bisphenol A; MET, methyl donor; BPA + MET, both bisphenol A and methyl donor supplementation in control diet. Different superscript letters within a row indicate significant difference (*p* < 0.05). At birth, 12 independent samples per treatment group (total 48); at weaning, six independent samples per treatment group (total 24).

**Table 6:** Effect of maternal methyl donor or bisphenol A supplementation during gestation on intestinal enzyme activity in newborn and weaning pigs.

	Treatment				*p*-Value		
	CON	BPA	MET	BPA + MET	BPA	MET	BPA × MET
Newborn Duodenum							
Lactase (U/mgprotein)	189.27 ± 31.20	185.04 ± 34.18	191.52 ± 36.03	187.91 ± 40.85	0.91	0.94	0.99
Maltase (U/mgprotein)	6.99 ± 1.18	6.76 ± 1.04	7.70 ± 0.99	7.42 ± 1.10	0.82	0.53	0.98
Sucrase (U/mgprotein)	1.75 ± 0.63	1.89 ± 0.77	2.43 ± 0.73	2.05 ± 0.98	0.88	0.60	0.75
Jejunum							
Lactase (U/mgprotein)	167.50 ± 28.77 ^b^	166.28 ± 27.43 ^b^	193.07 ± 30.33 ^a^	184.18 ± 30.33 ^a^	0.86	0.04	0.05
Maltase (U/mgprotein))	9.02 ± 1.10	8.91 ± 1.10	7.46 ± 1.16	8.34 ± 1.05	0.72	0.35	0.65
Sucrase (U/mgprotein)	1.73 ± 0.24 ^a^	1.00 ± 0.27 ^b^	1.80 ± 0.23 ^a^	1.10 ± 0.23 ^a,b^	<0.01	0.74	0.95
Ileum							
Lactase (U/mgprotein)	29.23 ± 5.03	24.33 ± 4.77	30.48 ± 4.55	31.27 ± 6.16	0.69	0.43	0.59
Maltase (U/mgprotein))	8.82 ± 1.10	5.86 ± 1.04	7.83 ± 0.95	8.62 ± 1.17	0.08	0.28	0.75
Sucrase (U/mgprotein)	1.22 ± 0.19	0.92 ± 0.18	0.91 ± 0.21	0.97 ± 0.22	0.58	0.52	0.38
Weaning Duodenum							
Lactase (U/mgprotein))	24.55 ± 10.67	32.45 ± 11.93	46.93 ± 10.67	35.13 ± 11.93	0.87	0.29	0.40
Maltase (U/mgprotein)	49.72 ± 9.44	48.05 ± 9.34	50.66 ± 12.19	45.75 ± 10.56	0.76	0.95	0.88
Sucrase (U/mgprotein)	7.71 ± 2.18 ^b^	8.61 ± 2.44 ^b^	15.84 ± 2.41 ^a^	12.35 ± 2.82 ^a^	0.75	0.02	0.51
Jejunum							
Lactase (U/mgprotein)	48.22 ± 8.98 ^b^	47.18 ± 11.59 ^b^	72.03 ± 8.20 ^a^	67.03 ± 11.59 ^a,b^	0.85	0.05	0.05
Maltase (U/mgprotein)	121.33 ± 23.82	102.37 ± 29.17	131.34 ± 23.32	143.15 ± 29.17	0.55	0.84	0.20
Sucrase (U/mgprotein)	51.61 ± 13.50 ^b^	43.53 ± 9.53 ^b^	81.90 ± 13.50 ^a^	69.98 ± 13.50 ^a,b^	0.07	0.01	0.22
Ileum							
Lactase (U/mgprotein)	5.99 ± 2.51	5.13 ± 2.49	6.64 ± 2.81	6.35 ± 2.61	0.56	0.50	0.97
Maltase (U/mgprotein)	80.81 ± 18.40	50.25 ± 18.40	83.58 ± 18.40	73.34 ± 16.79	0.27	0.48	0.58
Sucrase (U/mgprotein)	35.42 ± 11.98	29.20 ± 10.32	32.33 ± 11.42	39.14 ± 10.94	0.98	0.77	0.59

CON, control; BPA, bisphenol A; MET, methyl donor; BPA + MET, both bisphenol A and methyl donor supplementation in control diet. Different superscript letters within a row indicate significant difference (*p* < 0.05). At birth, 12 independent samples per treatment group (total 48); at weaning, six independent samples per treatment group (total 24).

**Table 7:** Effect of maternal methyl donor or bisphenol A supplementation during gestation on mRNA relative expression of nutrient transporters and disaccharidases in jejunum of newborn and weaning piglets.

	Treatment				*p*-Value			
	CON	BPA	MET	BPA + MET	BPA	MET	BPA × MET
Newborn							
*Slc7a9*	1.00 ± 0.32	0.86 ± 0.18	1.15 ± 0.18	0.97 ± 0.21	0.56	0.64	0.94
*Pept1*	1.00 ± 0.26 ^b^	0.54 ± 0.10 ^c^	2.35 ± 0.22 ^a^	1.07 ± 0.21 ^b^	<0.01	<0.01	0.11
*Sglt1*	1.00 ± 0.10 ^c^	1.16 ± 0.14 ^b,c^	2.37 ± 0.21 ^a^	1.59 ± 0.18 ^b^	0.10	<0.01	0.01
*Glut2*	1.00 ± 0.23	0.73 ± 0.17	1.40 ± 0.27	0.83 ± 0.18	0.09	0.31	0.54
*LCT*	1.00 ± 0.14 ^c^	1.24 ± 0.16 ^b,c^	2.90 ± 0.25 ^a^	1.89 ± 0.10 ^b^	0.67	<0.01	0.05
*SUC*	1.00 ± 0.13 ^b^	0.53 ± 0.08 ^c^	1.76 ± 0.12 ^a^	1.35 ± 0.06 ^a,b^	0.04	0.11	0.20
*MGAM*	1.00 ± 0.09	1.42 ± 0.13	1.62 ± 0.17	1.22 ± 0.20	0.34	0.29	0.57
Weaning							
*Slc7a9*	1.00 ± 0.11	0.97 ± 0.26	0.99 ± 0.17	1.02 ± 0.19	1.00	0.92	0.87
*Pept1*	1.00 ± 0.35 ^b,c^	0.40 ± 0.10 ^c^	1.84 ± 0.22 ^a^	1.37 ± 0.35 ^a,b^	0.05	<0.01	0.81
*Sglt1*	1.00 ± 0.18 ^b^	0.88 ± 0.29 ^b^	2.14 ± 0.40 ^a^	1.25 ± 0.31 ^a,b^	0.13	0.03	0.24
*Glut2*	1.00 ± 0.23	1.03 ± 0.16	1.42 ± 0.32	1.30 ± 0.34	0.87	0.24	0.79
*LCT*	1.00 ± 0.13 ^b^	0.85 ± 0.15 ^b^	1.73 ± 0.27 ^a^	0.93 ± 0.09 ^b^	0.71	0.04	0.91
*SUC*	1.00 ± 0.12	1.02 ± 0.09	1.15 ± 0.13	1.41 ± 0.07	0.95	0.89	0.09
*MGAM*	1.00 ± 0.09	1.18 ± 0.12	1.28 ± 0.16	1.20 ± 0.21	0.77	0.65	0.81

CON, control; BPA, bisphenol A; MET, methyl donor; BPA + MET, both bisphenol A and methyl donor supplementation in control diet; *Clc7a9*, amino acid transporter light chain, family 7, member 9; *Pept1*, peptide transporter 1; Sglt1, sodium/glucose co-transporter 1; *Glut2*, glucose transporter 2; *LCT*, lactase; *SUC*, sucrase-isomaltase (alpha-glucosidase); *MGAM*, maltase-glucoamylase. Data are normalized against *β-actin*, with results expressed relative to the control sample using the ^ΔΔ^*C*t method (where *C*t is the cycle threshold) with efficiency correction. Values are expressed as mean ± standard error; different superscript letters within a row indicate significant difference (*p* < 0.05). At birth, 12 independent samples per treatment group (total 48); at weaning, six independent samples per treatment group (total 24).

**Table 8:** Effect of maternal methyl donor or bisphenol A supplementation during gestation on the jejunum Pept1 gene DNA methylation level in newborn pigs.

CpG Site	Treatment				*p*-Value		
	CON	BPA	MET	BPA + MET	BPA	MET	BPA × MET
−18	0.54 ± 0.03 ^b^	0.46 ± 0.05 ^b^	0.70 ± 0.05 ^a^	0.53 ± 0.05 ^b^	0.02	0.02	0.33
−66	0.56 ± 0.03 ^b^	0.19 ± 0.04 ^c^	0.93 ± 0.04 ^a^	0.46 ± 0.04 ^b^	<0.01	<0.01	0.23
−93	0.87 ± 0.04 ^a,b^	0.35 ± 0.04 ^c^	0.94 ± 0.01 ^a^	0.78 ± 0.06 ^b^	<0.01	<0.01	<0.01
−149	0.76 ± 0.06	0.72 ± 0.02	0.77 ± 0.02	0.76 ± 0.02	0.49	0.54	0.68
−215	0.59 ± 0.07 ^b^	0.39 ± 0.05 ^c^	0.89 ± 0.04 ^a^	0.52 ± 0.03 ^b,c^	<0.01	<0.01	0.12
−278	0.87 ± 0.01	0.84 ± 0.02	0.87 ± 0.06	0.84 ± 0.02	0.35	0.94	1.00
−655	0.75 ± 0.03	0.50 ± 0.17	0.74 ± 0.03	0.75 ± 0.01	0.19	0.18	0.15
−672	0.79 ± 0.03	0.61 ± 0.21	0.78 ± 0.04	0.77 ± 0.02	0.38	0.49	0.44
−688	0.36 ± 0.07 ^b^	0.18 ± 0.06 ^c^	0.71 ± 0.09 ^a^	0.51 ± 0.14 ^b^	<0.01	<0.01	0.88
−704	0.44 ± 0.13	0.18 ± 0.14	0.39 ± 0.12	0.38 ± 0.13	0.32	0.59	0.37
−721	0.63 ± 0.04 ^b^	0.33 ± 0.04 ^c^	0.94 ± 0.03 ^a^	0.83 ± 0.04 ^a^	<0.01	<0.01	0.03
−774	0.76 ± 0.06	0.50 ± 0.17	0.73 ± 0.02	0.74 ± 0.03	0.22	0.29	0.17
−1159	0.19 ± 0.09	0.22 ± 0.13	0.19 ± 0.11	0.21 ± 0.14	0.83	0.95	0.99
−1169	0.93 ± 0.01	0.94 ± 0.01	0.93 ± 0.01	0.91 ± 0.01	0.85	0.26	0.34
−1203	0.13 ± 0.07	0.15 ± 0.08	0.15 ± 0.08	0.16 ± 0.09	0.87	0.82	0.99
−1252	0.15 ± 0.06	0.16 ± 0.01	0.20 ± 0.02	0.16 ± 0.03	0.72	0.54	0.54
−1352	0.77 ± 0.05 ^a^	0.38 ± 0.03 ^b^	0.87 ± 0.04 ^a^	0.43 ± 0.03 ^b^	<0.01	0.06	0.64
−1417	0.10 ± 0.06	0.09 ± 0.05	0.11 ± 0.06	0.07 ± 0.07	0.73	0.86	0.79
−1544	0.47 ± 0.05 ^b^	0.31 ± 0.05 ^c^	0.64 ± 0.05 ^a^	041 ± 0.04 ^a,b^	<0.01	0.01	0.45
−1694	0.60 ± 0.03	0.61 ± 0.02	0.61 ± 0.02	0.59 ± 0.02	0.88	0.80	0.52
−1705	0.28 ± 0.06 ^c^	0.20 ± 0.05 ^c^	0.67 ± 0.04 ^a^	0.47 ± 0.07 ^b^	0.03	<0.01	0.35
−1718	0.05 ± 0.01	0.06 ± 0.02	0.07 ± 0.02	0.05 ± 0.02	0.82	0.94	0.34
−1771	0.34 ± 0.03 ^a^	0.23 ± 0.03 ^b^	0.33 ± 0.05 ^a,b^	0.26 ± 0.03 ^a,b^	0.02	0.72	0.48
−1795	0.92 ± 0.01	0.92 ± 0.02	0.91 ± 0.02	0.93 ± 0.03	0.72	0.90	0.72
−1908	0.33 ± 0.04	0.34 ± 0.01	0.32 ± 0.02	0.31 ± 0.01	0.76	0.36	0.61

CON, control; BPA, bisphenol A; MET, methyl donor; BPA + MET, both bisphenol A and methyl donor supplementation in control diet. Different superscript letters within a row indicate significant difference (*p* < 0.05). Four independent samples per treatment group (total 16).

## Supplementary Materials

The following are available online at www.mdpi.com/2072-6643/9/5/423/s1, Table S1: Effect of maternal methyl donor or bisphenol A supplementation during gestation on body weight in newborn and weaning pigs.
