# Supplementary material for: Maternal Methyl Donor Supplementation during Gestation Counteracts the Bisphenol A-Induced Impairment of Intestinal Morphology, Disaccharidase Activity, and Nutrient Transporters Gene Expression in Newborn and Weaning Pigs
Source: Nutrients. 2017 Apr 26;9(5):423. doi: 10.3390/nu9050423 (PMC5452153; doi:10.3390/nu9050423)
Supplement: Supplementary file 1 [file nutrients-09-00423-s001.pdf]

Table S1. Effect of maternal methyl donor or bisphenol A supplementation during gestation on body weight in newborn and weaning pigs.

	**Treatment**				***p*-value**		
	**CON**	**BPA**	**MET**	**BPA + MET**	**BPA**	**MET**	**BPA × MET**
No. litters, *n*	13	12	13	12			
No. piglets, *n*	12.86 ± 0.89	12.62 ± 0.56	12.25 ± 1.05	14.55 ± 0.90	0.24	0.45	0.15
Birth weight, kg	1.24 ± 0.02 ^{b}	1.38 ± 0.03 ^{a}	1.34 ± 0.03 ^{a}	1.33 ± 0.03 ^{a}	0.01	0.66	<0.01
Weaning weight, kg	6.51 ± 0.35 ^{c}	7.06 ± 0.51 ^{b}	7.74 ± 0.24 ^{a,b}	7.14 ± 0.37 ^{a,b}	0.95	0.09	0.16

CON, control; BPA, bisphenol A; MET, methyl donor; BPA + MET, both bisphenol A and methyl donor supplementation in control diet. No. litters, *n*: the average number of litters in this group; No. piglets, *n*: the average number of piglets per litter in this group; Birth weight: the average weight of all newborn piglets in this group; Weaning weight: the average weight of all weaning piglets in this group. Within a row, means with different superscript letters are significantly different ($p < 0.05$).
